# Insight into metabolic sensors of nitrosative stress protection in *Phytophthora infestans*

**DOI:** 10.3389/fpls.2023.1148222

**Published:** 2023-07-20

**Authors:** Joanna Gajewska, Jolanta Floryszak-Wieczorek, Arkadiusz Kosmala, Dawid Perlikowski, Marek Żywicki, Ewa Sobieszczuk-Nowicka, Howard S. Judelson, Magdalena Arasimowicz-Jelonek

**Affiliations:** ^1^Department of Plant Ecophysiology, Institute of Experimental Biology, Faculty of Biology, Adam Mickiewicz University in Poznań, Poznań, Poland; ^2^Department of Plant Physiology, Poznań University of Life Sciences, Poznań, Poland; ^3^Institute of Plant Genetics, Polish Academy of Sciences, Poznań, Poland; ^4^Department of Computational Biology, Institute of Molecular Biology and Biotechnology, Faculty of Biology, Adam Mickiewicz University in Poznań, Poznań, Poland; ^5^Department of Plant Physiology, Faculty of Biology, Adam Mickiewicz University in Poznań, Poznań, Poland; ^6^Department of Microbiology and Plant Pathology, University of California, Riverside, Riverside, CA, United States

**Keywords:** reactive nitrogen species, nitric oxide dioxygenase, peroxiredoxins, nitrosative stress, *Phytophthora infestans*, late blight

## Abstract

*Phytophthora infestans*, a representative of phytopathogenic oomycetes, have been proven to cope with redundant sources of internal and host-derived reactive nitrogen species (RNS). To gain insight into its nitrosative stress resistance mechanisms, metabolic sensors activated in response to nitrosative challenge during both *in vitro* growth and colonization of the host plant were investigated. The conducted analyses of gene expression, protein accumulation, and enzyme activity reveal for the first time that *P. infestans* (avirulent MP946 and virulent MP977 toward potato cv. Sarpo Mira) withstands nitrosative challenge and has an efficient system of RNS elimination. The obtained data indicate that the system protecting *P. infestans* against nitric oxide (NO) involved the expression of the nitric oxide dioxygenase (Pi-NOD1) gene belonging to the globin family. The maintenance of RNS homeostasis was also supported by an elevated S-nitrosoglutathione reductase activity and upregulation of peroxiredoxin 2 at the transcript and protein levels; however, the virulence pattern determined the expression abundance. Based on the experiments, it can be concluded that *P. infestans* possesses a multifarious system of metabolic sensors controlling RNS balance *via* detoxification, allowing the oomycete to exist in different micro-environments flexibly.

## 1 Introduction

Both host organisms and fungal or fungal-like pathogens can synthesize nitric oxide (NO), which, as a cross-kingdom signal molecule, is engaged in the initiation, coordination, and transfer of molecular information on various stimuli. Considering plant–pathogen interactions, plants initiate the NO burst already during the first minutes after the pathogen has been identified to trigger multiple modes of defense events to create a potent antimicrobial environment (Floryszak-Wieczorek et al., 2007). In turn, phytopathogens generate NO during infection structure formation, which was evidenced in fungi, e.g., *Blumeria graminis* (Prats et al., 2008), *Oidium neolycopersici* (Piterková et al., 2011), *Magnaporthe oryzae* (Samalova et al., 2013), and *Fusarium graminearum* (Ding et al., 2020), as well as oomycetes such as *Bremia lactucae* (Sedlářová et al., 2011) and *Phytophthora infestans* (Izbiańska et al., 2019). Although the kinetics and intensity of NO generation depend on the type of plant resistance, the colonization of plant tissues by pathogens often results in an overproduction of NO and NO‐derived compounds including peroxynitrite (ONOO^−^). This creates a local hot spot of boosted and pathophysiological levels of reactive nitrogen species (RNS), defined as nitrosative stress (Arasimowicz-Jelonek and Floryszak-Wieczorek, 2014). In consequence, pathogens during the infection process must cope with their own redundant NO and plant-derived RNS. In these circumstances, pathogen survival is strictly dependent on its metabolic equipment to quench or reset the boosted NO signal and counteract nitrosative stress. Owing to a high reactivity of RNS towards various biomolecules, deficiency in the NO detoxification activities may result in cell dysfunction or damage, similarly to the case of oxidative stress. Thus, the elements of the NO/RNS detoxification system constitute a sophisticated adaptation mechanism to the persistence of huge RNS amounts mounted by internal and external sources, and it might also be implicated in phytopathogen virulence (Arasimowicz-Jelonek and Floryszak-Wieczorek, 2014).

Detoxification activity is essential to fungal or fungal-like pathogens not only to escape the host-induced nitrosative stress. Adverse abiotic environmental conditions can also provoke uncontrolled accumulation of RNS in diverse pathogen structures (Gajewska et al., 2020). Moreover, the maintenance of NO homeostasis must be sensibly regulated to exert its signaling functions during microbial development. It is well documented that NO endogenously produced by microorganisms is an important regulatory molecule involved in the switch between developmental phases of the phytopathogens, sporulation, germination, fruiting body formation, and production of secondary metabolites (Cánovas et al., 2016; Zhao et al., 2020).

The balance of NO in the cellular environment can be governed by several NO detoxification systems. Nitric oxide dioxygenase (NOD), which belongs to the globin family, has been the best characterized so far in the model microorganisms belonging to fungi and bacteria (Liu et al., 2000; de Jesús-Berríos et al., 2003; Philippe et al., 2003; Ullmann et al., 2004; Hromatka et al., 2005; Turrion-Gomez et al., 2010; Lapp et al., 2014). Nitric oxide dioxygenases are enzymes that efficiently mediate the reaction of deoxygenation using NAD(P)H to integrate two atoms from O_2_ into NO, forming nitrate (Poole, 2020). Importantly, the NO dioxygenase activity is exhibited by flavohemoglobins (Fhbs). Proteins coded by the *Fhb* genes could be located in the cytosol and mitochondria to ensure mechanisms for NO depletion in particular cellular compartments (Zhou et al., 2011). Fhbs proteins were identified in several important fungal phytopathogens (Boccara et al., 2005). Importantly, to date, the specific activity of Fhb for *in vivo* NO decomposition has been confirmed only for *Bcfhg1* in *Botrytis cinerea* (Turrion-Gomez et al., 2010). Although deletion of the *Fhb* gene in model human pathogens attenuated their virulence (de Jesús-Berríos et al., 2003; Ullmann et al., 2004), the deletion of *Bcfhg1* did not affect the pathogenicity of the microorganism (Turrion-Gomez et al., 2010). Also, in *M. oryzae*, an Fhb-encoding gene *MoFHB1* was found to be dispensable for pathogen virulence (Zhang et al., 2022). A similar phenomenon has been described in the plant pathogenic bacterium *Erwinia chrysanthemi*, whereby deletion of the Fhb coding gene created mutants unable to host infection (Boccara et al., 2005).

The other system contributing to cellular protection against nitrosative stress employs S-nitrosoglutathione reductase (GSNOR). This GSH-dependent bi-functional enzyme conserved from bacteria to humans is able to reduce S-nitrosoglutathione (GSNO) to form GSSG and ammonia, as well as detoxify formaldehyde (Tillmann et al., 2015). Cloning and biochemical characterization of AnGSNOR from the model filamentous fungus *Aspergillus nidulans* confirmed that fungal GSNOR could efficiently regulate NO bioactivity for signaling purposes and it consequently enhances cellular resistance to nitrosative stress (Zhou et al., 2012; Zhou et al., 2016). In addition, other proteins have also been found to be involved in the detoxification of NO in the model fungi. The porphobilinogen deaminase encoded by *hemC* facilitated RNS-tolerant fungal growth by promoting the activity of the Fhbs (Zhou et al., 2012). Moreover, the *ntpA* gene coding a cysteine-rich 23-amino-acid peptide may undergo S-nitrosylation to generate nitrosothionein and consequently remove NO (Zhou et al., 2013).

The fungus-like oomycete *P. infestans* (Mont.) de Bary is one of the most important plant pathogens worldwide causing late blight disease in the *Solanaceae* family, particularly in potato and tomato (Dong and Zhou, 2022). Global annual yield losses and control costs of potato crops were estimated at ca. 6.5 billion US dollars worldwide (Savary et al., 2019; Adolf et al., 2020). Importantly, in recent decades, a significant shift in the infection potential of *P. infestans* has been observed as a result of increasing genetic variation, pathogenicity, and pathogen migration (Fry et al., 2015; Michalska et al., 2016). In consequence, the rapid evolution and adaptive capacity of *P. infestans* are the causes of the still unsatisfactory progress in the battle against phytopathogens (Forbes, 2012). Additionally, the molecular mechanisms exploited by *P. infestans* to establish host colonization and adaptation to new micro-environments are still poorly understood. Our last research revealed that the fungus-like organism uses the formation of RNS as an inherent element of the pathogen adaptation strategy to survive in the host environment (Izbiańska et al., 2019). To date, there is no experimental knowledge concerning the metabolic adjustment of the oomycetes that allow the regulation of innate RNS and/or counteract host-induced nitrosative stress during the infection process. In view of the above, the aim of the present study was to evidence that *P. infestans* has the ability to withstand and decompose high levels of RNS and indicate metabolic components engaged in the cellular protection of *P. infestans* against nitrosative stress under *in vitro* and *in planta* conditions. Our experimental approach involved avirulent (avr MP946) and virulent (vr MP977) *P. infestans* isolates (in reference to the potato genotype “Sarpo Mira”), creating a useful background for the identification of RNS-mediated metabolic events favorable for pathogen virulence and successful host colonization. As documented, *P. infestans* cope with a nitrosative challenge by activating RNS scavenging system that includes Pi-NOD1, GSNOR, and PRX2; however, the virulent isolate showed potent induction of GSNOR gene expression and enzyme activity. Moreover, potato-vr *P. infestans* interaction was accompanied by an early upregulation of *Pi-NOD1* gene expression.

## 2 Materials and methods

### 2.1 Pathogen culture

*P. infestans* (Mont.) de Bary—the avirulent isolate MP946 (race 1.3.4.7.10.11) and the virulent MP977 (race 1.2.3.4.6.7.10) in reference to the potato cv. Sarpo Mira (carrying the *R* genes: *R3a*, *R3b*, *R4*, *Rpi-Smira1*, and *Rpi-Smira2*)—was provided by the Plant Breeding Acclimatization Institute (IHAR), Research Division in Młochów, Poland. *In vitro* and *in planta* studies were performed according to Izbiańska et al. (2019) mainly at the Department of Plant Ecophysiology, Adam Mickiewicz University for 3 years (2020–2023). Briefly, for *in vitro* studies, the pathogen was grown for 9 days in the dark on a cereal-potato agar medium with an addition of dextrose (control culture) or the medium was additionally supplemented with RNS modulators as described in *Section 2.2*. For *in planta* studies: potato plants cv. Sarpo Mira (from the Potato Gene Bank—Plant Breeding and Acclimatization Institute—IHAR in Bonin, Poland) were inoculated by spraying with 3 ml of a freshly prepared suspension of sporangia and zoospores (5.0 × 10^5^ sporangia per ml) and incubated in sterile boxes for 9 days at 16°C and 95% relative humidity in the dark. Sporangia of *P. infestans* were obtained by washing 9-day-old cultures with cold distilled water and zoospores were released by incubating the sporangia in water at 4°C for 30 min.

The hyphae growing *in vitro* or *in planta* were manually collected and directly analyzed or frozen in liquid nitrogen and stored at −80°C. Hyphae *in planta* were additionally isolated by dipping the infected tissues in 5% cellulose acetate, letting the acetone evaporate, and stripping the cellulose acetate film off according to Both et al. (2005).

### 2.2 Nitrosative stress, hyphal growth, and spore germination

For the *in vitro* growth experiment, the following RNS donors were added to the growing medium on the first day of the *P. infestans* culture: sodium nitroprusside (SNP; Merck) at concentrations of 100 µM, 250 µM, 500 µM, and 1 mM; S-nitrosoglutathione (GSNO; Sigma-Aldrich) at concentrations of 100 µM, 200 µM, 350 µM, and 500 µM; and 3-morpholinosydnonimine (SIN-1; Calbiochem) at concentrations of 250 µM, 500 µM, 1 mM, and 5 mM. Additionally, 500 µM light-exposed SNP as well as RNS scavengers, i.e., 200 μM 2-phenyl-4,4,5,5,-tetramethylimidazoline-1-oxyl 3-oxide (PTIO; Sigma-Aldrich) and 100 μM ebselen (Cayman Chemicals), were used. Control cultures were treated with sterile water. Donors of RNS are pharmacologically active compounds that release RNS and mimic endogenous RNS-related effects after application to biological systems. All used donors exhibit differential RNS-releasing ability in aqueous solutions. The solution’s half-life of NO donors is 7 h for GSNO and 12 h for SNP (Floryszak-Wieczorek et al., 2006), whereas SIN-1 spontaneously decomposes in two steps, releasing superoxide anion and NO in an aqueous solution and yielding a continuous source of ONOO^−^ over several hours (Izbiańska et al., 2019). Importantly, SNP to release NO requires light or single-electron reduction by reducing agents present in the biological systems such as ascorbates, hemoproteins, thiols, NADH, and NADPH (Floryszak-Wieczorek et al., 2006). To ensure conditions for the release of various RNS from donors, after treatment, the pathogen culture was left for 5 h under constant light, including control.

Radial growth of *P. infestans* was measured every day for a period of 9 days. All treatments were analyzed at least in triplicate, and for each biological replicate, five technical replicates were prepared.

To mimic nitrosative stress under *in vitro* conditions in further analyses, 500 µM SNP, 350 µM GSNO, and 5 mM SIN-1 were selected. Additionally, the scavenger of NO (200 μM PTIO) and ONOO^−^ (100 μM ebselen) were used to estimate RNS-dependent effects. Material for molecular and biochemical analyses was collected on the 9th day of the culture. In the case of *in planta* transcriptional analysis of Pi-NOD, the material was also collected in the following hours after potato inoculation with *P. infestans* spore suspension.

### 2.3 Assessment of spore germination

As described in *Section 2.1*, the freshly prepared spore suspension from the control and RNS modulator-treated hyphae was used for the calculation of indirect germinating spores. After spores were released by incubation at 4°C, the number of germinating spores was determined. For the counting, 100 µl of spore suspension was transferred to microscopic glass covered with 1% agar, and after 24 h, microscopic counting was performed.

### 2.4 Measurement of cell death

Cell death, indicated as a loss of plasma membrane integrity, was estimated on the basis of Evans Blue uptake according to Ederli et al. (2009). Hyphae (0.150 g) of *P. infestans* were incubated for 20 min in 0.25% (w/v) Evans Blue (Sigma-Aldrich). The stained hyphae were then washed twice for 15 min in distilled water and homogenized with 1.5 ml of 1% (w/v) SDS. After a centrifugation at 12,000 rpm for 15 min, the Blue Evans uptake, indicating cell death, was measured spectrophotometrically at λ = 600 nm.

### 2.5 Nitric oxide measurement

Nitric oxide was monitored with a PGSTAT 30 universal electrochemical analyzer (EcoChemie, Utrecht, the Netherlands). The concentration of NO liberated from 100 μM spermine NONOate (Cayman Chemicals) was measured by differential pulse amperometry with an NO selective needle-type electrode prepared as described by Floryszak-Wieczorek and Arasimowicz-Jelonek (2016). Pathogen extracts were prepared from hyphae (0.250 g) growing *in planta*, as well as non-treated and GSNO-treated *in vitro* cultures in 500 µl of 100 mM sodium phosphate buffer, pH 7.4, using mortars and pestles. After protein concentration measurement, the extracts were diluted to reach a final protein concentration of 1 mg/ml. The current was recalculated into concentration units on the basis of a calibration curve. NO scavenging measurements were performed in gas-tight vials by adding to spermine NONOate solution 1 ml of the extract containing 1 mg of *P. infestans* proteins in 100 mM sodium phosphate buffer, pH 7.4. The temperature of the mixture was kept at 24°C in a water bath and the time-dependent changes of the NO signal were recorded.

### 2.6 Peroxynitrite reductase activity

Peroxynitrite-mediated oxidation of dihydrorhodamine 123 to rhodamine was followed by adding 1 μM SIN-1 as ONOO^−^ donor to 1 ml of the reaction mixture containing 50 mM potassium phosphate buffer, pH 7.0, 20 μM dihydrorhodamine 123 (Sigma-Aldrich), 20 μM DTPA (Sigma-Aldrich), and 100 µl of *P. infestans* extract prepared in 50 mM potassium phosphate buffer, pH 7.0. Rhodamine was then measured at 500 nm as reported previously (Bryk et al., 2000). BSA, which has no peroxynitrite reductase activity, was used as a negative control.

### 2.7 Gene expression measurement

Hyphae of *P. infestans* were frozen in liquid nitrogen and stored at −80°C until use. The RNA was isolated from 0.150 g of frozen sample using TriReagent (Sigma-Aldrich). The obtained RNA was purified with the use of the Deoxyribonuclease Kit (Sigma-Aldrich). For the reverse transcription, 1 µg of RNA was processed with the Reverse Transcription Kit (Thermo Scientific Fermentas) according to the manufacturer’s instructions. The real-time PCR reactions were performed on a Rotor-Gene 6000 thermocycler (Corbett Life Science, Qiagen). The reaction mixture contained 0.1 µM of each primer (listed in **Supplementary Table S1**), 1 µl of 5× diluted cDNA, 5 µl of Power SYBR Green PCR Master mix (Applied Biosystems), and DEPC-treated water to a total volume of 10 µl. The PCR reaction initiated denaturation at 95°C for 5 min. Subsequent stages included 50 cycles consisting of 10 s at 95°C, 20 s at 53°C, and 30 s at 72°C. The reaction was finalized by denaturation at a temperature rising from 72°C to 95°C by 1°C every 5 s. The reaction specificity and CT values for individual samples were determined using the real-time PCR Miner Program (Zhao and Fernald, 2005). The *P. infestans S3a* gene was selected as a reference in the *P. infestans* gene expression measurement according to Gajewska et al. (2020). The relative gene expression was calculated using the Pfaffl mathematical model (Pfaffl, 2001).

### 2.8 Cloning of Pi-NOD cDNA

The cDNA sequence encoding the *P. infestans* nitric oxide dioxygenase (Pi-NOD1) mRNA was obtained from the genome of two *P. infestans* (Mont.) de Bary isolates—the avirulent isolate MP946 (race 1.3.4.7.10.11) and the virulent MP977 (race 1.2.3.4.6.7.10)—using the PCR method. Primers for the protein coding region were designed based on *P. infestans* T30-4 nitric oxide dioxygenase (Pi-NOD1) (PITG_22661) mRNA; complete CDS was obtained from the GenBank database, accession number XM_002909124.1. The forward primer was [ATGGCTCCCAACCAACAGAC] and the reverse primer was [CACCAAGCCAGTTCGAGACT]. The resulting PCR product was purified using the QIAEXII Gel Extraction Kit (Qiagen) and ligated into the pGEM-T Easy vector (Promega). Next, the *Escherichia coli* strain XL1 Blue was transformed with the ligation mixture. The selected clones (X-Gal and IPTG) carrying an appropriate PCR product were sequenced (Molecular Biology Techniques Laboratory, Faculty of Biology, Adam Mickiewicz University, Poznań). The obtained sequences were processed with the BioEdit software (ver. 7.2.5). According to the consensus sequence obtained for both strains, a homologous region encoding peptide, (NH2)-SHHRVAGATKGGAPPGC-(amidated), was selected. The peptide was used further as the antigen to produce the specific antibody in the rabbit host (Agrisera).

### 2.9 Western blot analysis (Pi-NOD and PRX2)

Protein accumulation profiles of Pi-NOD and PRX2 were analyzed. Total proteins were extracted using the Hurkman and Tanaka (1986) protocol with slight modifications described earlier by Lechowicz et al. (2020), whereas Western blot assay was performed as described by Pawłowicz et al. (2012). For immunodetection, specific (Agrisera) or commercial (Abcam) antibodies were applied. Briefly, the antibody against Pi-NOD was produced as described in *Section 2.8*. In turn, for PRX2 detection, the commercial rabbit polyclonal antibody was applied. The Pi-NOD antibody was diluted at 1:4,000, while the PRX2 antibody was diluted at 1:2,000. Antigen–antibody complexes were detected using a secondary anti-rabbit IgG–horseradish peroxidase conjugate (Sigma-Aldrich) diluted 1:30,000 and incubated for 1 h for Pi-NOD and 1:20,000 for 2 h for PRX2 detection, respectively. Chemiluminescent substrates Westar Supernova (Cyanagen) and ChemiDoc™ Touch Igmagin System (Bio-Rad) were used to visualize the results. The intensities of visualized bands were estimated using the ImageJ software. The band intensities were first normalized with respect to the amounts of proteins detected in the gels stained with Coomassie Blue. Furthermore, to eliminate technical errors between blots, the obtained intensity data for experimental samples were divided by the intensity of internal control standard, which was present in every blot in the same amount and was used as a normalization marker. The internal control standard consisted of the total proteins extracted in the control growth conditions from the mixture of virulent and avirulent isolates.

### 2.10 GSNOR activity

The GSNOR (EC 1.2.1.46) activity was determined according to the procedure proposed by Barroso et al. (2006) with minor modifications described by Arasimowicz-Jelonek et al. (2014). Fresh hyphae (0.5 g) were homogenized in 0.1 M Tris-HCl buffer, pH 7.5, containing 0.2% Triton X-100 (v/v), 10% glycerol (v/v), 0.1 mM EDTA, and 2 mM DTT and centrifuged at 27,000 *g* for 25 min. Supernatants were passed through Sephadex G-25 gel filtration columns (Illustra NAP-10 from GE Healthcare), then immediately through Amicon Ultra 3K Filters (Millipore). One milliliter of the assay reaction mixture contained 0.5 mM EDTA, 0.2 mM NADH, 0.4 mM GSNO, and 30 µl of enzyme extract in 25 mM Tris-HCl buffer, pH 8.0. The reaction was run at 25°C and initiated with an addition of GSNO to reach the final 0.4 mM concentration in the reaction mixture. NADH oxidation was determined at 340 nm and rates of NADH consumed at min^−1^ were calculated using an extinction coefficient of 6,220 M^−1^ cm^−1^.

### 2.11 Quantification of total S-nitrosothiols

Total SNO contents were determined by chemiluminescence using a Sievers® Nitric Oxide Analyzer NOA 280i (GE Analytical Instruments, Boulder, CO, USA) according to Chaki et al. (2009) with minor modifications described by Arasimowicz-Jelonek et al. (2014). Fresh hyphae (0.5 g) were homogenized in Tris-HCl 0.1 M buffer, pH 7.5 (1:4, w/v), containing 100 µM DTPA, 1 mM EDTA, 1 mM EGTA, 1 mM PMSF, 0.1 mM neocuproine, 3.5% (w/v) PVPP, 0.25% (v/v) Triton X-100, and centrifuged at 3,000 *g* for 15 min. The supernatants were incubated with 10 mM NEM (N-ethylmaleimide) for 15 min at 4°C and subsequently two aliquots were prepared for each sample. To remove nitrite, one aliquot was incubated for 15 min with 10 mM sulfanilamide at 4°C. To eliminate nitrite and decompose SNOs, the next aliquot was treated with 10 mM sulfanilamide and 7.3 mM HgCl_2_ for 15 min at 4°C. The difference between the detected signals obtained from these aliquots demonstrated the total SNO content.

### 2.12 Phylogenetic analysis and protein sequence alignment

The phylogenetic tree of Pi-NOD1 orthologs has been obtained from Ensembl Protist database (GeneTree ID: EPrGT00050000005075) and simplified for clarity. The methodology of the tree construction can be accessed at Ensembl https://protists.ensembl.org/info/genome/compara/homology_method.html.

The alignments of *Phytophthora* Pi-NOD1 protein sequences have been constructed using the COBALT sequence alignment tool and Pi-NOD1 protein sequences obtained from the Ensembl database representing translated sequences of the longest transcripts of the orthologous genes (Howe et al., 2019). The transcript IDs are presented in brackets on **Supplementary Figure S2**.

### 2.13 Statistical analysis

All results are based on three biological replicates derived from three independent experiments. For each experiment, means of the obtained values (*n* = 9) were calculated along with standard deviations. All statistical analyses were performed in STATISTICA 10.0 software (StatSoft, Tulsa OK, USA). To estimate the statistical significance between means, the data were analyzed with the use of one-way analysis of variance (ANOVA) followed by Dunnett’s test at the level of significance α = 0.05 or α = 0.01.

## 3 Results

### 3.1 *P. infestans* withstands high levels of exogenous RNS

**Figure 1:**
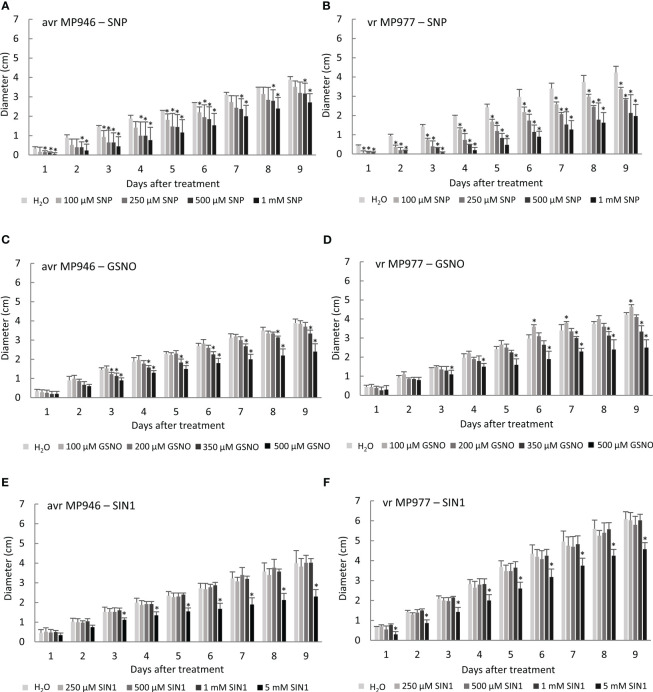
The effect of various concentrations of reactive nitrogen species (RNS) donors on avr/vr *Phytophthora infestans* during 9 days of *in vitro* growth. Radial growth of **(A)** avr MP946 and **(B)** vr MP977 on medium supplemented with 0, 100, 250, 500 µM, and 1 mM of sodium nitroprusside (SNP); **(C)** avr MP946 and **(D)** vr MP977 on medium supplemented with 0, 100, 200, 350, and 500 µM of S-nitrosoglutathione (GSNO); and **(E)** avr MP946 and **(F)** vr MP977 on medium supplemented with 0, 250, 500 µM, 1 mM, and 5 mM of 3-morpholinosydnonimine (SIN1). The results are averages from three independent experiments (*n* = 15) ± SD. Asterisks indicate values that differ significantly from the non-treated samples (control) *P. infestans* culture at each time point (day) at *p* < 0.01 (*).

To determine a tolerance threshold of *P. infestans* to NO and ONOO^−^, the growth of the culture treated with different concentrations of various RNS donors, i.e., SNP, GSNO, and SIN-1, was measured every day for a period of 9 days (**Figure 1**). Exogenous NO added as SNP significantly reduced hyphal growth already after the first day of the oomycete culture (**Figures 1A, B**). The growth area gradually decreased with an increasing SNP concentration, which was especially observed in vr MP977 *P. infestans*. On the 9th day of the avr MP946 and vr MP977 cultures, the growth was significantly reduced by the two highest SNP concentrations, compared to the control pathogen culture (**Figures 1A, B**). Application of GSNO at the lower concentration, i.e., 100 µM, even induced growth of hyphae in vr *P. infestans* MP977 by ca. 10% (**Figure 1D**). The significant growth inhibition by ca. 20% and 40% was noted at 350 µM and 500 µM GSNO in both isolates. A peroxynitrite donor, SIN-1, did not affect *P. infestans* growth in the concentration range from 250 µM to 1 mM (**Figures 1E, F**). Growth reduction was only observed at 5 mM SIN-1 and was 24% and 42% in vr MP977 and avr MP946, respectively.

**Figure 2:**
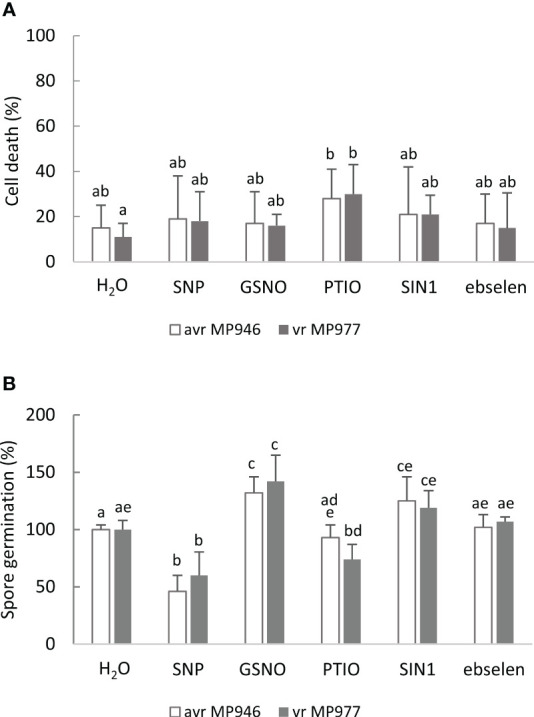
The effect of selected RNS donors (500 µM SNP, 350 µM GSNO, and 5 mM SIN1) and scavengers (200 µM 2-phenyl-4,4,5,5,-tetramethylimidazoline-1-oxyl 3-oxide, PTIO, and 100 µM ebselen) on the 9th day on **(A)** avr/vr *Phytophthora infestans* cell death and **(B)** spore germination. The amount of germinating spores was determined 24 h after preparation of the spore suspension. Values represent means ± SD of three biological replicates derived from three independent experiments (*n* = 9). Columns marked with the same letter are not significantly different (Dunnett’s test) at *p* < 0.05.

To mimic nitrosative stress conditions in further analyses, RNS donor doses resulting in a significant growth inhibition of both *P. infestans* isolates were selected. These included 500 µM SNP, 350 µM GSNO, and 5 mM SIN-1. It should be noted that the specific scavengers significantly attenuated the RNS-mediated growth inhibitory effect (**Supplementary Figure S1**), and none of the selected doses of NO modulators affected the cell death rate of the avr/vr pathogen culture (**Figure 2A**). Moreover, the process of indirect spore germination was accelerated *via* GSNO and SIN-1 donors by approximately 36% and 20%, respectively. In contrast, spore germination obtained from the SNP-treated culture of avr MP946 and vr MP977 was inhibited by ca. 54% and 40%, respectively (**Figure 2B**).

### 3.2 *P. infestans* shows *in vitro* ability to scavenge RNS

**Figure 3:**
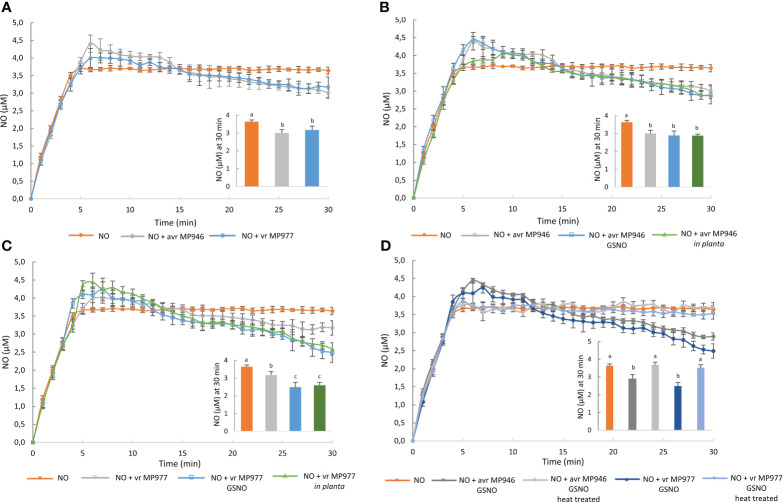
Nitric oxide scavenging activity of avr/vr *Phytophthora infestans* on the 9th day. Nitric oxide levels liberated from 100 μM spermine NONOate were measured amperometrically in the absence or presence of avr/vr *P. infestans* for 30 min. **(A)** The comparison of NO decomposition activity between avr MP946 and vr MP977. The effect of nitrosative stress supplied as 350 µM GSNO and *in planta* growth conditions on NO scavenging activity of **(B)** avr MP946 and **(C)** vr MP977. **(D)** Nitric oxide scavenging activity of avr/vr *P. infestans* exposed to 350 µM GSNO and next thermally inactivated. The NO level noted at 30 min was presented as the embedded chart. Values represent means ± SD of three biological replicates derived from three independent experiments (*n* = 3). Columns marked with the same letter are not significantly different (Dunnett’s test) at *p* < 0.05.

To explore the ability of *P. infestans* to RNS elimination, first NO scavenging activity of the pathogen was assessed by the electrochemical method. The NO donor at a concentration of 100 μM spermine NONOate was used as a background, since the amount of NO in the solution was found to reach a steady-state level of 3.7 μM NO after 7 min (Zhu et al., 2018). Next, the ability of avr/vr *P. infestans* hyphal extracts to degrade NO liberated from the donor compound was monitored (**Figure 3**). Real-time NO detection revealed that avr/vr *P. infestans* scavenged NO under *in vitro* conditions, but no difference was noted between the analyzed isolates with respect to the kinetics of NO detoxification (**Figure 3A**). Nitrosative stress and *in planta* conditions accelerated the NO scavenging activity of vr MP977 *P. infestans* since NO concentration significantly decreased at 30 min of the pathogen incubation with the donor spermine NONOate. It decreased from the background level of 3.7 μM to 3.17 μM and 2.6 μM in the control and GSNO-treated/*in planta* cultures, respectively (**Figure 3C**). The thermally inactivated extract of avr/vr *P. infestans* hyphae had no ability to NO scavenging (**Figure 3D**).

**Figure 4:**
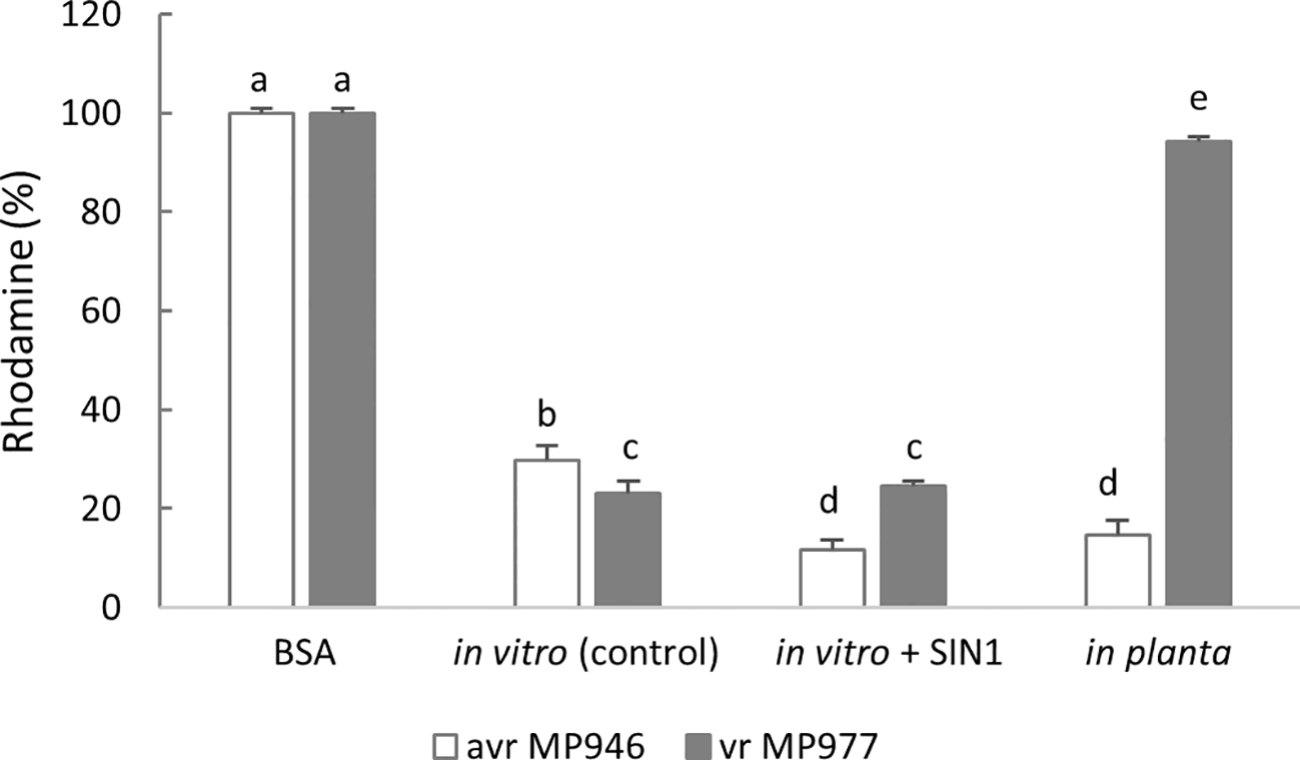
Peroxynitrite detoxification activity of avr/vr *Phytophthora infestans* on the 9th day measured as peroxynitrite-mediated oxidation of dihydrorhodamine 123 to rhodamine. The 100% value was defined as the amount of rhodamine formed in the absence of protein. The activity was measured in avr MP946 and vr MP977 growing in the following conditions: *in vitro*-control, *in vitro* + 5 mM SIN1, and *in planta*. Values represent means ± SD of three biological replicates derived from three independent experiments (*n* = 9). Columns marked with the same letter are not significantly different (Dunnett’s test) at *p* < 0.05.

Next, to verify if *P. infestans* is able to eliminate ONOO^−^, an assay of ONOO^−^ detoxification activity was performed using peroxynitrite-mediated oxidation of dihydrorhodamine 123 to rhodamine. The approach allowed us to determine a peroxynitrite reductase activity and assumed that the 100% value reflected the amount of rhodamine formed in the absence of protein. As observed, both isolates of non-treated *P. infestans* cultures were able to avoid the ONOO^−^-mediated oxidation of dihydrorhodamine 123, demonstrating an effective ONOO^−^ detoxification activity (**Figure 4**). An elevated activity expressed as a low percentage of rhodamine formation was noted in avr MP946 *P. infestans* growing in the presence of SIN-1 and *in planta.* In turn, in vr MP977 *P. infestans* exposed to nitrative conditions, ONOO^−^ detoxification activity was at the same level as in the control conditions (**Figure 4**).

### 3.3 Nitrosative conditions induce the *Pi-NOD* gene in *P. infestans*

Oomycota includes four main orders *Saprolegniales, Pythiales, Peronosporales*, and *Albuginales* (Beakes et al., 2012; McCarthy and Fitzpatrick, 2017). Moreover, the order *Peronosporales* includes *Phytophthora* species and represents the main terrestrial and plant pathogenic lineage (Beakes and Thines, 2017).

Since *P. infestans* withstands high levels of exogenous RNS and has the ability to decompose them, the next experimental steps allowed us to identify sensors engaged in the metabolic adjustment of the phytopathogen to nitrosative stress.

**Figure 5:**
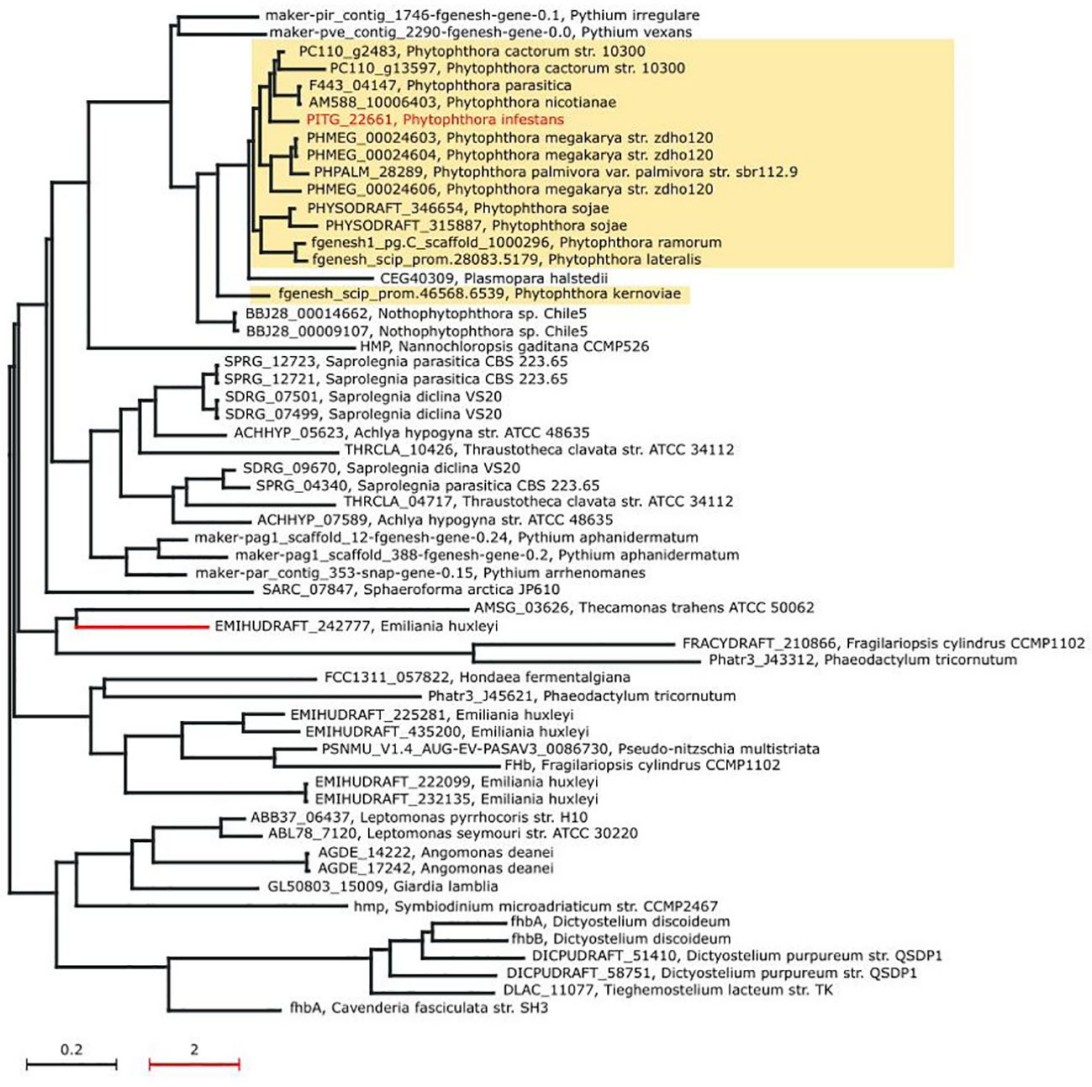
Maximum likelihood phylogenetic tree of the nitric oxide dioxygenase (Pi-NOD1) orthologs. The phylogenetic distance of the red branch has been scaled down 10× to increase the clarity of the tree. For each node of the tree, the accession number of NOD gene ortholog and the organism name have been provided.

First, phylogenetic analysis illustrated that nitric oxide dioxygenase of *Phytophthora*, *Nothophytophthora* sp. Chile 5, *Plasmopara halstedii*, and *Pythium* species represent a separate phylogenetic group. It is one of four major groups that are relatively separated from each other (**Figure 5**). *Nothophytophthora* is a sister group with *Phytophthora* (Jung et al., 2017) and *Pythium* species was previously included together with *Phytophthora* to the same order (Ho, 2018). Other groups are formed by Pi-NOD proteins from (i) *Saprolegnia*, *Achlya*, *Pythium*, and *Thraustotheca* species; (ii) *Emiliania*, *Fragilariopsis*, and *Pseudo-nitzschia* species; (iii) *Leptomonas*, *Angomonas*, *Giardia*, *Symbiodinium*, *Dictyostelium*, *Cavenderia*, and *Tieghemostelium* species. Interestingly, sequences of Pi-NOD from other plant pathogenic species, like *Pythium irregulare*, *Pythium vexans*, and *Plasmopara halstedi*, are very similar to *Phytophthora*. After closer inspection, protein sequences among different *Phytophthora* are highly conserved (55.2%–99.79% pairwise identity), and the observed sequence changes do not affect physicochemical properties (hydrophaty) (**Supplementary Figure S2**). To provide more detailed insight into conservation of Pi-NOD among different *Phytophthora*, the alignment of Pi-NOD protein sequences was performed. This analysis allows one to omit low confidence orthologs, including partial sequences, which have been included by Ensembl Compara for construction of the phylogenetic tree presented in **Figure 5**. The result revealed that the protein sequences among different *Phytophthora* are highly conserved (77.07%–99.79% pairwise identity), and the observed sequence changes do not affect physicochemical properties (hydropathy) (**Supplementary Figure S2**). Moreover, similar to the previously studied fungal plant pathogen *B. cinerea*, *P. infestans* contains only a single gene copy of NOD enabling reliable studies of the Pi-NOD protein using diverse genetic techniques. Thus, the Pi-NOD1 from *P. infestans* is a good experimental model for all *Phytophthora*, as well as for some other pathogenic Stramenopiles.

**Figure 6:**
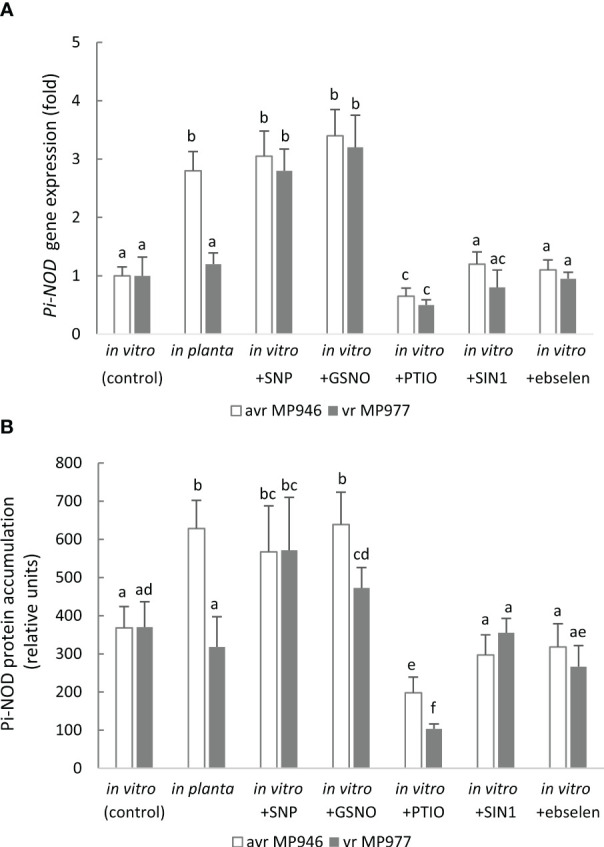
**(A)** Transcript and **(B)** protein levels of *Phytophthora infestans* nitric oxide dioxygenase (Pi-NOD1) in avr MP946 and vr MP977 growing in *in vitro-*control, *in vitro-*nitrosative stress (in the presence of selected RNS modulators: 500 µM SNP, 350 µM GSNO, 5 mM SIN1, 200 µM PTIO, and 100 µM ebselen) on the 9th day, and *in planta* conditions on the 9th day. The transcript accumulation level of *P. infestans* S3a was used as the reference. The gene expression was determined using the RT-qPCR method and the protein concentration was measured using the 2D-Quant Kit (GE Healthcare). Values represent means ± SD of three biological replicates derived from three independent experiments (*n* = 9). Columns marked with the same letter are not significantly different (Dunnett’s test) at *p* < 0.05.

In order to determine whether exposure of the oomycete to RNS influences the expression of Pi-NOD1 that belongs to the globin family, the *Pi-NOD1* gene expression during *in vitro* pathogen growth was investigated (**Figure 6A**). Application of 500 µM SNP and 350 µM GSNO provoked a ca. threefold increase in *Pi-NOD1* transcript accumulation, while NO scavenging resulted in a significant gene downregulation in both isolates in comparison to the non-treated control cultures. Moreover, *Pi-NOD1* gene expression was also monitored during the early hours of potato-avr/vr *P. infestans* interactions (**Supplementary Figure S3**). As found, *Pi-NOD1* was upregulated especially in vr MP977 *P. infestans* up to 48 hpi, while during the later hpi, the gene expression decreased. Next, the production of anti-Pi-NOD allowed us to analyze the accumulation pattern of Pi-NOD1 in response to RNS (**Figure 6B**). It should be noted that the antibody recognized two bands and only the higher one (approximately 40 kDa instead of a theoretical band at 49 kDa) was considered for the analysis. NO donors enhanced contents of Pi-NOD1 protein in *P. infestans* structures in relation to the control, and the tendency was observed in both isolates exposed to SNP and in avr MP946 growing in the presence of GSNO. Moreover, *in planta* hyphal growth resulted in a higher accumulation of both the Pi-NOD1 transcript and protein as compared to *in vitro* conditions.

It should be noted that the ONOO^−^ donor and its scavenger did not cause any significant changes at the Pi-NOD1 transcript and protein levels, indicating that Pi-NOD1 induction is specifically NO-dependent (**Figures 6A, B**).

### 3.4 GSNOR activity affects the metabolic status of NO in *P. infestans*

**Figure 7:**
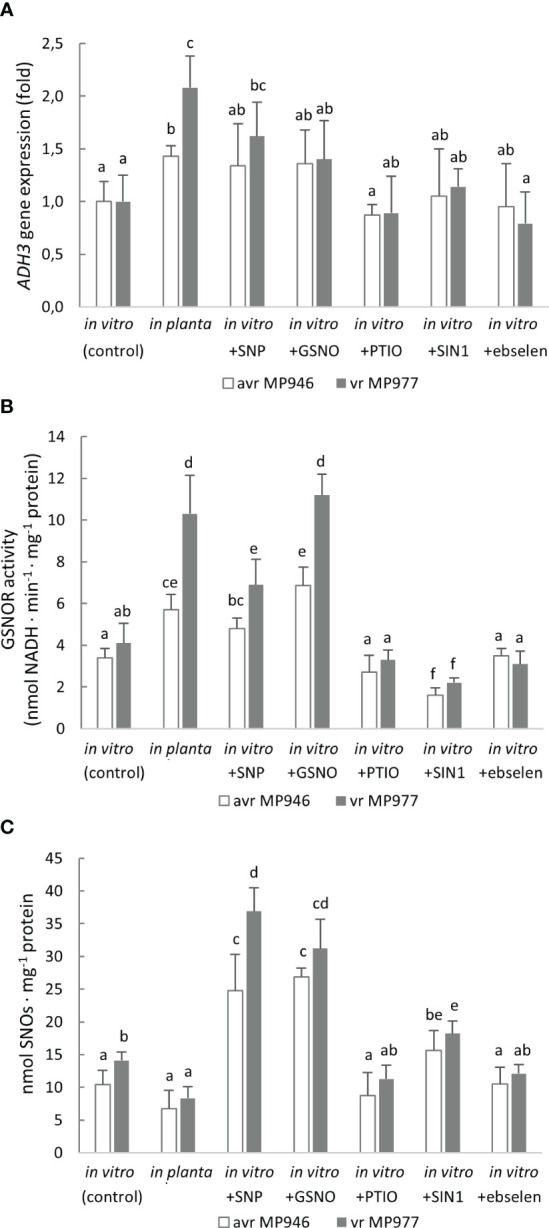
**(A)** Alcohol dehydrogenase (ADH3) gene expression, **(B)** S-nitrosoglutathione reductase activity, and **(C)** total content of S-nitrosothiols in avr MP946 and vr MP977 *P. infestans* growing in *in vitro*-control, *in vitro*-nitrosative stress (in the presence of the selected RNS modulators: 500 µM SNP, 350 µM GSNO, 5 mM SIN1, 200 µM PTIO, and 100 µM ebselen) on the 9th day, and *in planta* conditions on the 9th day. The gene expression was determined using the RT-qPCR method. Values represent means ± SD of three biological replicates derived from three independent experiments (*n* = 9). Columns marked with the same letter are not significantly different (Dunnett’s test) at *p* < 0.05.

A Zn-dependent medium-chain class III alcohol dehydrogenase (ADH3) is recognized as GSNOR, which is considered another element of the defense mechanism against nitrosative stress. Moreover, *P. infestans* contains only a single gene copy of ADH3. Therefore, to verify whether GSNOR controls cellular NO homeostasis in *P. infestans*, *ADH3* transcript accumulation and GSNOR enzyme activity were determined (**Figures 7A, B**). Although *in planta* conditions significantly enhanced *ADH3* gene expression in both pathogen isolates, the highest, ca. twofold increase in *ADH3* transcript accumulation was noted in vr MP977. *In vitro* nitrosative and *in planta* growing conditions also evoked induction of the GSNOR activity in *P. infestans*. A strong, approximately twofold upregulation of GSNOR activity was noted in vr MP977 hyphae treated with GSNO and under *in planta* conditions. In turn, treatment of avr MP946 with SNP, GSNO, and the *in planta* phase elevated the activity from 40% to 100%. Although the NO donor-treated *P. infestans* cultures revealed a significantly elevated GSNOR activity, the level of SNOs was ca. threefold higher (**Figure 7C**). In contrast, an *in planta* increase of GSNOR activity correlated with a diminished pool of SNOs.

### 3.5 *PRX2* is an RNS-responsive gene in *P. infestans*

**Figure 8:**
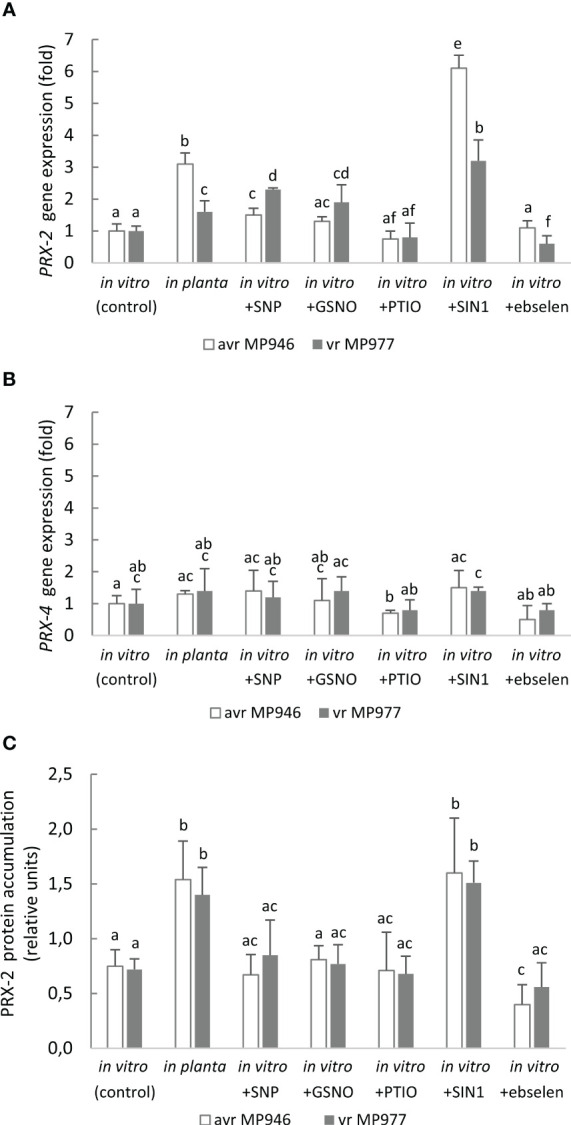
Transcriptional analysis of **(A)** *PRX2*, **(B)** *PRX4*, and **(C)** content of PRX2 protein in avr MP946 and vr MP977 *P. infestans* growing in *in vitro*-control, *in vitro*-nitrosative stress (in the presence of the selected RNS modulators: 500 µM SNP, 350 µM GSNO, 5 mM SIN1, 200 µM PTIO, and 100 µM ebselen) on the 9th day, and *in planta* conditions on the 9th day. The gene expression was determined using the RT-qPCR method. Values represent means ± SD of three biological replicates derived from three independent experiments (*n* = 9). Columns marked with the same letter are not significantly different (Dunnett’s test) at *p* < 0.05.

To further explore the metabolic adjustment that could favor *P. infestans* survival under a high level of RNS, the expression of *PRX2* and *PRX4* was analyzed (**Figures 8A, B**). These encode peroxiredoxins, which have a potential peroxynitrite reductase activity (Bryk et al., 2000; Romero-Puertas et al., 2007). However, only *PRX2* was found to be ONOO^−^-responsive. The abundance of the *PRX2* transcript was remarkably high in response to SIN-1 treatment and reached a ca. six- and threefold increase in avr MP946 and vr MP977, respectively (**Figure 8A**). Also, *in planta* conditions significantly enhanced the *PRX2* transcript accumulation by approximately 210% and 60% in avr MP946 and vr MP977, respectively. A similar pattern of PRX2 protein accumulation was observed in *P. infestans* growing under *in vitro* and nitrosative stress conditions (**Figure 8C**). The abundance of PRX2 protein showed the highest, ca. twofold, accumulation in *P. infestans* exposed to SIN-1 and grown *in planta*.

## 4 Discussion

Previous research on avr/vr *P. infestans*–potato pathosystems revealed that both adversaries are able to activate NO synthesis already during the first minutes of the plant–pathogen interaction, and that both might employ NO for their own benefit (Floryszak-Wieczorek and Arasimowicz-Jelonek, 2016; Izbiańska et al., 2019). The presented *in vitro* study revealed that avr/vr *P. infestans* can persist under relatively high RNS concentrations. Most of the used RNS donor concentrations were within the tolerance limit noted for *P. infestans* since they inhibited the growth of hyphae by up to 50%. Importantly, the pathogen cell death rate remained unaltered, while the process of spore germination was even accelerated in response to *in vitro* nitrosative challenge. Only in the case of SNP an inhibition of spore germination was observed, which could be due to toxic cyanogenic groups released during the donor decomposition. Additionally, our experiment revealed that *P. infestans* has a high level of resistance to ONOO^−^. The donor SIN-1 that provides a continuous source of ONOO^−^ (Kelm et al., 1997; Delledonne et al., 2001) did not affect *P. infestans* cell death rate even at a concentration of 5 mM. While the pathogen survives high concentrations of RNS, an elevated level of these reactive molecules is known to impair plant disease responses. For example, loss of *GSNOR1* in Arabidopsis leads to an elevated level of NO correlated with enhanced DNA methylation and reduced expression of stress-responsive genes (Rudolf et al., 2021). Likewise, *PR1* expression was reduced and delayed in the GSNOR1-deficient Arabidopsis mutant *gsnor1-3* (Rudolf et al., 2021).

It was earlier reported that taxonomically distinct filamentous ascomycete fungi can also persist in high NO concentrations, although NO application inhibited mycelium growth, sporulation, and spore germination of the postharvest horticultural pathogens *Aspergillus niger*, *Monilinia fructicola*, and *Penicillium italicum* (Lazar et al., 2008). Moreover, the treatment of *Colletotrichum coccodes* spores with 100 µM SNP significantly inhibited their germination and development (Wang and Higgins, 2005). There are also few *in vitro* experiments linking the fungal nitrosative response with the promotion of reproductive processes. For example, SNP treatment of the wild-type strain and the conidiation-deficient mutant of *Coniothyrium minitans* improved conidial yield and restored conidia production, respectively (Gong et al., 2007). Moreover, the application of the L-arginine as the NO synthesis promoter positively regulated urediniospore germination in *Puccinia striiformis* f. sp. *Tritici* (Yin et al., 2016). A high level of resistance to ONOO^−^ was earlier observed in human pathogenic bacteria, whereby treatment with 1 mM ONOO^−^ resulted in nearly 100 % viability and was dependent on the strain virulence pattern (Yu et al., 1999; Barth et al., 2009; Piacenza et al., 2019). Plants also showed peroxynitrite resistance since the exposure of soybean cells to exogenous ONOO^−^ did not result in cell death even at the concentration of 5 mM SIN-1, whereas in animal cells, a dose-dependent cell death was observed even at 1 µM of ONOO^−^ prepared from a stock solution in NaOH (Delledonne et al., 2001). Importantly, the ability of the virulent versus avirulent strains of microbes to cope with RNS may greatly differ both qualitatively and quantitatively (Piacenza et al., 2019).

The successful colonization of the potato tissues by vr *P. infestans* involves a strong overproduction of NO and NO-derived molecules associated with pathogen existence in the *in planta* nitrosative behavior. As previously shown, (1) the level of NO and ONOO^−^ in vr *P. infestans* was relatively high during both *in vitro* and *in planta* growth; (2) increased production of both RNS during *in planta* growth was localized in mature sporangia, which may favor zoospores releasing and providing fast host colonization; (3) in vr *P. infestans*, only a slight difference in the total protein pool undergoing nitration was observed during late blight development compared to *in vitro* growth, indicating high pathogen adaptation to both host and its own RNS (Izbiańska et al., 2019). Since the excess of NO might be toxic to eukaryotic cells, the balance between RNS production and the quenching state determines therefore a key axis of the host–pathogen interaction (Warris and Ballou, 2019). Thus, the ability of cells to cope with NO is essential in determining the biological fate of NO synthesis and in consequence for survival under nitrosative stress conditions. Insight into the protection system against nitrosative stress in the oomycete plant pathogens showed that *P. infestans* withstands high concentrations of NO and its derivative due to the capability to eliminate active RNS. The phenomenon can be a consequence of an adaptation strategy to the environment enriched with NO from both internal (pathogen) and external (host) sources (Arasimowicz-Jelonek et al., 2014). As reviewed recently by Taheri (2022), in plants, some hemoglobins maintain homeostasis of NO and, as a consequence, provide tolerance to different unfavorable conditions. Moreover, some heme-containing enzymes are engaged in ROS/RNS metabolism and participate in defense response mechanisms against both biotic and abiotic stresses (Taheri, 2022). In bacterial and fungal microorganisms, the maintenance of the NO balance is possible *via* several metabolic sensors; however, a central sensor system to metabolize NO and counteract nitrosative stress involves Fhb, an efficient NO dioxygenase (Gardner et al., 2006). As indicated by Turrion-Gomez et al. (2010), studying NO dioxygenases helps provide insight into the biological functions of NO and the mechanisms involved in its detoxification in various systems. Genome sequencing of *P. infestans* T30-4 revealed the NO dioxygenase (*Pi-NOD1*) gene belonging to the globin family, which has not been previously studied in any detail. Notably, the *Pi-NOD1* phylogeny does not follow the organismal phylogeny. The distances between *NOD* genes reflect rather the environmental lifestyle of the organisms. NOD proteins can be potentially involved in host adaptation to high levels of NO and might be essential targets for treatment. The current study found that *P. infestans* contains only a single gene copy of NOD, which is expressed during *in vitro* avr/vr *P. infestans* growth, while exposure to exogenous NO enhanced its expression. *In planta* conditions induced Pi-NOD1 at the transcript and protein levels only in avr *P. infestans* MP946. In vr *P. infestans* MP977 under the *in planta* phase, the expression pattern of Pi-NOD gene and protein was parallel with those observed during *in vitro* growth. The results suggest that the physiological functions of Pi-NOD1 in *P. infestans* could be more related to the modulation of endogenous NO levels during development, rather than to the modulation of NO during the infection process. Related to our observation, a relatively high level of *Bcfhg1* expression, a functional *B. cinerea* Fhb, was detected only during the very early stages of tomato leaf infection (at 8 hpi) associated with spore germination (Turrion-Gomez et al., 2010). According to Anta-Fernández et al. (2022), NO formed during germination exerts a repressive effect on the process and the flavohemoglobin activity could provide a positive regulation mechanism that allows spore germination to progress. During most of the infection process, *B. cinerea* is in contact with elevated NO, since the expression of *Bcfhg1* after 8 hpi was maintained at a low level (Turrion-Gomez et al., 2010). It should be noted that *B. cinerea* as a necrotroph exhibits physiological differences in comparison with biotrophic or hemibiotrophic plant pathogens such as *P. infestans*. The maintenance of an NO-rich host environment may have important consequences in the establishment and progress of disease, since pathogen-derived NO could enrich plant cells and contribute to the hypersensitive cell death, facilitating subsequent tissue colonization (Turrion-Gomez and Benito, 2011; Arasimowicz-Jelonek and Floryszak-Wieczorek, 2014). Importantly, our finding of *Pi-NOD1* gene expression during the early hours of potato-avr/vr *P. infestans* interactions suggests that *Pi-NOD1*-dependent NO decomposition could operate during the biotrophic phase of the pathogen, while the later stages of successful potato colonization especially in the case of vr *P. infestans* were not associated with the gene expression, and NO depletion might be supported by another, yet unknown system. It cannot be ruled out that an enhanced *Pi-NOD1* gene expression during the contact with the host tissues could be a result of a host-derived nutritional stimulus, since nitrate and nitrite have been shown to induce the Fhb expression coding gene in several species of fungi and bacteria (Ullmann et al., 2004; Arai et al., 2005).

Flexibility and multiplicity of defense and offensive strategies seem to be essential in pathogenic microorganisms that exist both free in the environment and in association with a host (Missall et al., 2004). Thus, regulation of NO homeostasis at the cellular level can be supported by GSNOR activity that modulates the transnitrosylation equilibrium between the most common low-molecular weight S-nitrosothiol—GSNO—and S-nitrosylated proteins. Additionally, the enzyme may control the cell redox state affecting levels of NADH and GSH (Jahnová et al., 2019). Our study revealed that both nitrosative and *in planta* conditions elevated GSNOR activity in the oomycete, which was particularly evident in vr *P. infestans* MP977. As documented, a boosted GSNOR activity associated with the decreased content of the S-nitrosylated protein pool was noted only in the *in planta* growth indicating that GSNOR actively controls NO/SNO homeostasis during the infection process (the progress of disease). It was earlier documented that in hemibiotrophic rice blast fungus *M. oryzae*, an S-(hydroxymethyl)glutathione dehydrogenase (MoSFA1) specifically catalyzes the reduction of GSNO and is involved in redox homeostasis (Fernandez and Wilson, 2014; Zhang et al., 2015). Moreover, MoSFA1 activity is crucial for proper development, and it contributes to virulence (Zhang et al., 2015). Notably, for double deletion of *MoSFA1* and *MoFHB1*, nitrosative stress was more severe and no further reduction in pathogenicity was found compared with the *MoSFA1* mutant (Zhang et al., 2022). In turn, in *Sclerotinia sclerotiorum*, formaldehyde dehydrogenase *SsFdh1* seems to be crucial for overcoming the NO-based host defense and increase the necrotroph pathogenicity (Zhu et al., 2019). In contrast, the abolished GSNO-consuming activity in a human pathogen *C. neoformans* GNO1 mutant did not affect the fungal growth under nitrosative challenge, while it did not reduce its virulence. Furthermore, in flavohemoglobin-null mutants, GNO1 was able to partially compensate to promote survival of *C. neoformans* in the infected host milieu (de Jesús-Berríos et al., 2003). It is worth pointing that the role of GSNOR in plant immunity is very complex (Jahnová et al., 2019). Loss-of-function mutations in GSNOR or reduction of GSNOR transcript abundance compromise multiple modes of plant disease resistance, while overexpression of this gene conveys increased disease resistance (Hussain et al., 2019). Contrary results have been also reported, as transgenic plants with decreased GSNOR gene expression showed activation of the pathogenesis-related (PR-1) gene and enhanced basal resistance (Rustérucci et al., 2007).

To counteract high RNS levels, microorganisms may also employ peroxiredoxins, which are highly reactive and abundant Cys-based peroxidases. These may play dual roles of H_2_O_2_ sensor and scavenger (Mir et al., 2015; Rocha et al., 2018). Importantly, PRXs catalytically reduce ONOO^−^ *in vitro*. In turn, modulation of PRXs expression affects ONOO^−^-mediated cytotoxicity *in vivo*, indicating a physiological role of these enzymes in ONOO^−^ reduction (Trujillo et al., 2008). Expression profiles of *P. infestans PRXs* showed that *PRX2* is responsive to both exogenous ONOO^−^ and the host environment. What is important, the virulence pattern determined the expression abundance. In contact with the plant host, avr *P. infestans* MP946 showed a higher PRX2 transcript and protein accumulation correlated in time with the pathogen ONOO^−^ detoxification activity. This upregulation can be a result of an accelerated ONOO^−^ formation observed in the isolate during the avr *P. infestans* switch to the *in planta* phase. In contrast, the ONOO^−^ level in vr *P. infestans* was relatively high during both *in vitro* and *in planta* growth (Izbiańska et al., 2019). It is worth pointing that NO itself is able to regulate PRX activity and thus ONOO^−^ content (Romero-Puertas et al., 2007). Moreover, nitroproteome analysis indicated that PRX2 undergoes nitration in *P. infestans* (Izbiańska et al., 2019). Thereby, PRX2 might fine-tune the damaging and signaling effects of ONOO^−^ in the oomycete.

## 5 Conclusion

To summarize, *P. infestans*, a representative of the oomycetes, withstands nitrosative challenge and possesses RNS elimination capacity. To gain insight into its nitrosative stress resistance mechanisms, metabolic sensors activated in response to nitrosative challenge during both *in vitro* growth and colonization of the host plant were investigated. As documented, a scavenging system protecting the aggressor against RNS can involve Pi-NOD1, GSNOR, and PRX2. However, the *P. infestans* virulence pattern determines qualitative and quantitative differences in coping with RNS. Thus, a goal of future studies is the functional characterization of *P. infestans* genes encoding the prime candidates of nitrosative stress resistance, as they are likely to be essential for pathogen virulence.

## Data availability statement

The original contributions presented in the study are included in the article/**Supplementary Material**. Further inquiries can be directed to the corresponding author.

## Author contributions

Conceptualization (conceived the ideas and designed the experiment), MA-J and JF-W. Formal analysis and investigation, JG, AK, DP, and MŻ. Validation, MA-J and JF-W. Writing—original draft preparation, JG and MA-J. Writing—review and editing, MA-J, JF-W, AK, MŻ, ES-N, and HJ. Supervision and project administration, MA-J. All authors contributed to the article and approved the submitted version.

## Supplementary material

Supplementary Figure 1 The effect of RNS modulators on avr/vr *Phytophthora infestans* during 9 days of *in vitro* growth. Radial growth of **(A)** avr MP946 and **(B)** vr MP977 on medium supplemented with 0, 500 µM of SNP, and 500 µM light-exposed SNP; **(C)** avr MP946 and **(D)** vr MP977 on medium supplemented with 0, 350 µM of GSNO, and 200 µM of PTIO; **(E)** avr MP946 and **(F)** vr MP977 on medium supplemented with 0, 5 mM of SIN1, and 100 µM of ebselen. The results are averages from three independent experiments (*n* = 15) ± SD. Columns marked with the same letter are not significantly different (Dunnett’s test) at *p* < 0.05

Supplementary Figure 2 Protein sequence alignment of the *Phytophthora* Pi-NOD1 high-confidence orthologs from the Ensembl Compara database. The upper panel represents the alignment colored according to the difference from amino acid consensus at the given alignment column: dark red represents highly differing positions; grey denotes similar. The bottom panel represents the alignment colored by hydropathy scale: blue represents hydrophilic amino acids, and red represents hydrophobic.

Supplementary Figure 3 *In planta Pi-NOD* gene expression analysis in the following hours post inoculation. The level of expression of *Pi-NOD1* at each time point (hpi) is given relative to the level of expression of *S3a*, considered a constitutively expressed gene. The gene expression was determined using the RT-qPCR method. Columns marked with the same letter are not significantly different (Dunnett’s test) at *p* < 0.05.

Supplementary Figure 4 Representative Western blot of **(A)** PiNOD and **(B)** PRX2 protein accumulation.

Supplementary Figure 5 Representative SDS-PAGE of proteins stained with Coomassie Brilliant Blue preformed in parallel during (A) PiNOD and (B) PRX2 immunodetection. Fifteen micrograms of protein of each sample was loaded per lane.

Supplementary Table 1 Sequences of primers used for the real-time PCR reaction.
