# Supplementary figures and images for: Insight into metabolic sensors of nitrosative stress protection in Phytophthora infestans
Source: Front Plant Sci. 2023 Jul 20;14:1148222. doi: 10.3389/fpls.2023.1148222 (PMC10399455; doi:10.3389/fpls.2023.1148222)

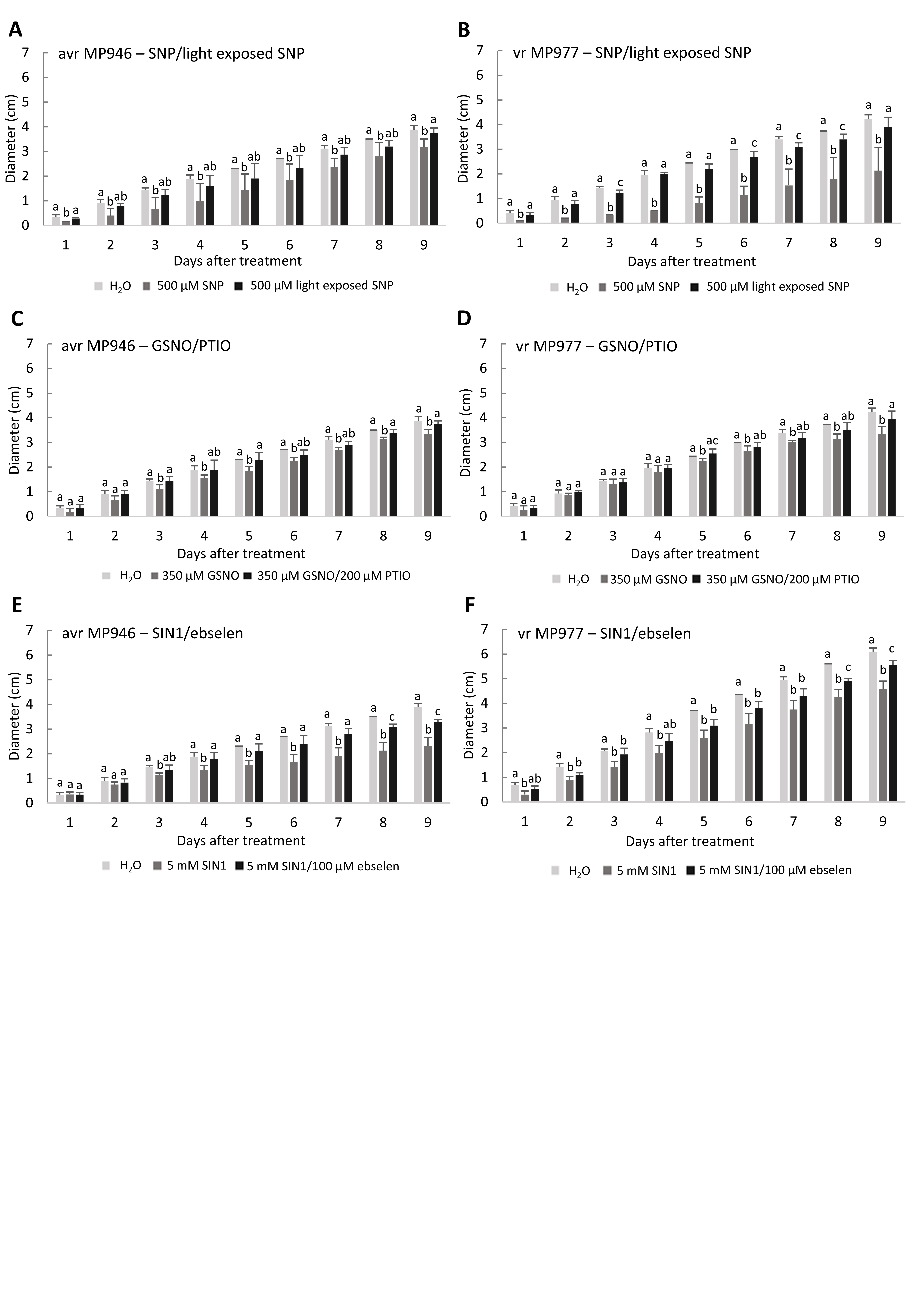

Supplement: Supplementary Figure 1 — The effect of RNS modulators on avr/vr Phytophthora infestans during 9 days of in vitro growth. Radial growth of (A) avr MP946 and (B) vr MP977 on medium supplemented with 0, 500 µM of SNP, and 500 µM light-exposed SNP; (C) avr MP946 and (D) vr MP977 on medium supplemented with 0, 350 µM of GSNO, and 200 µM of PTIO; (E) avr MP946 and (F) vr MP977 on medium supplemented with 0, 5 mM of SIN1, and 100 µM of ebselen. The results are averages from three independent experiments (n = 15) ± SD. Columns marked with the same letter are not significantly different (Dunnett’s test) at p < 0.05 [file Image_1.tif]

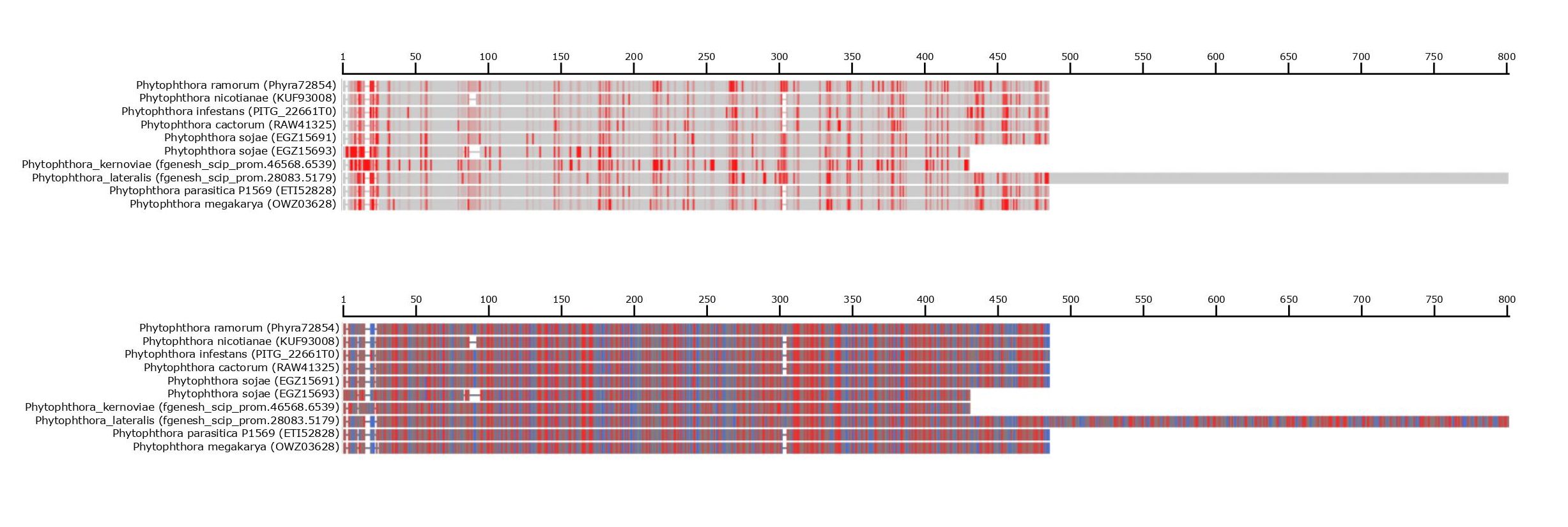

Supplement: Supplementary Figure 2 — Protein sequence alignment of the Phytophthora Pi-NOD1 high-confidence orthologs from the Ensembl Compara database. The upper panel represents the alignment colored according to the difference from amino acid consensus at the given alignment column: dark red represents highly differing positions; grey denotes similar. The bottom panel represents the alignment colored by hydropathy scale: blue represents hydrophilic amino acids, and red represents hydrophobic. [file Image_2.jpeg]

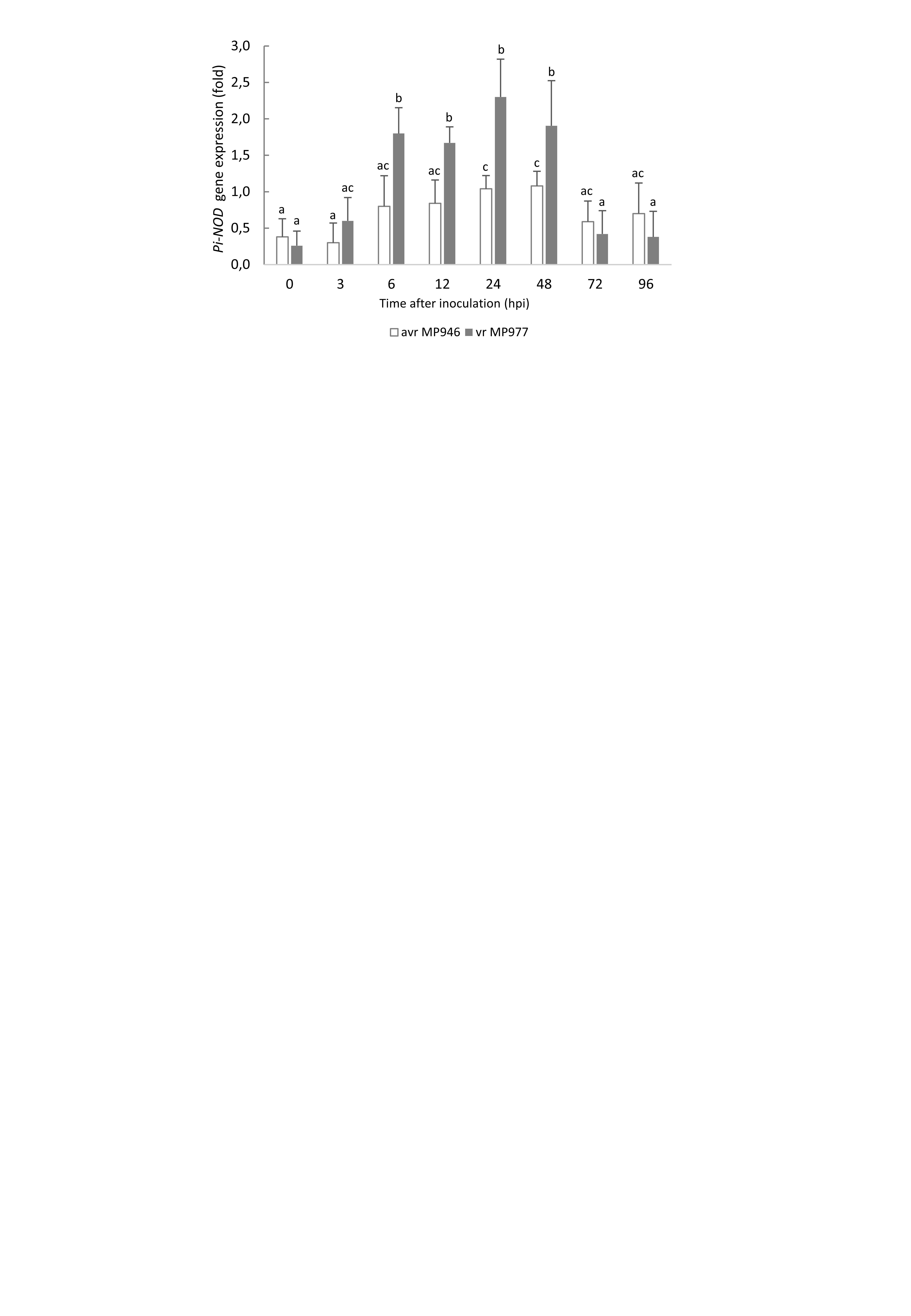

Supplement: Supplementary Figure 3 — In planta Pi-NOD gene expression analysis in the following hours post inoculation. The level of expression of Pi-NOD1 at each time point (hpi) is given relative to the level of expression of S3a, considered a constitutively expressed gene. The gene expression was determined using the RT-qPCR method. Columns marked with the same letter are not significantly different (Dunnett’s test) at p < 0.05. [file Image_3.tif]

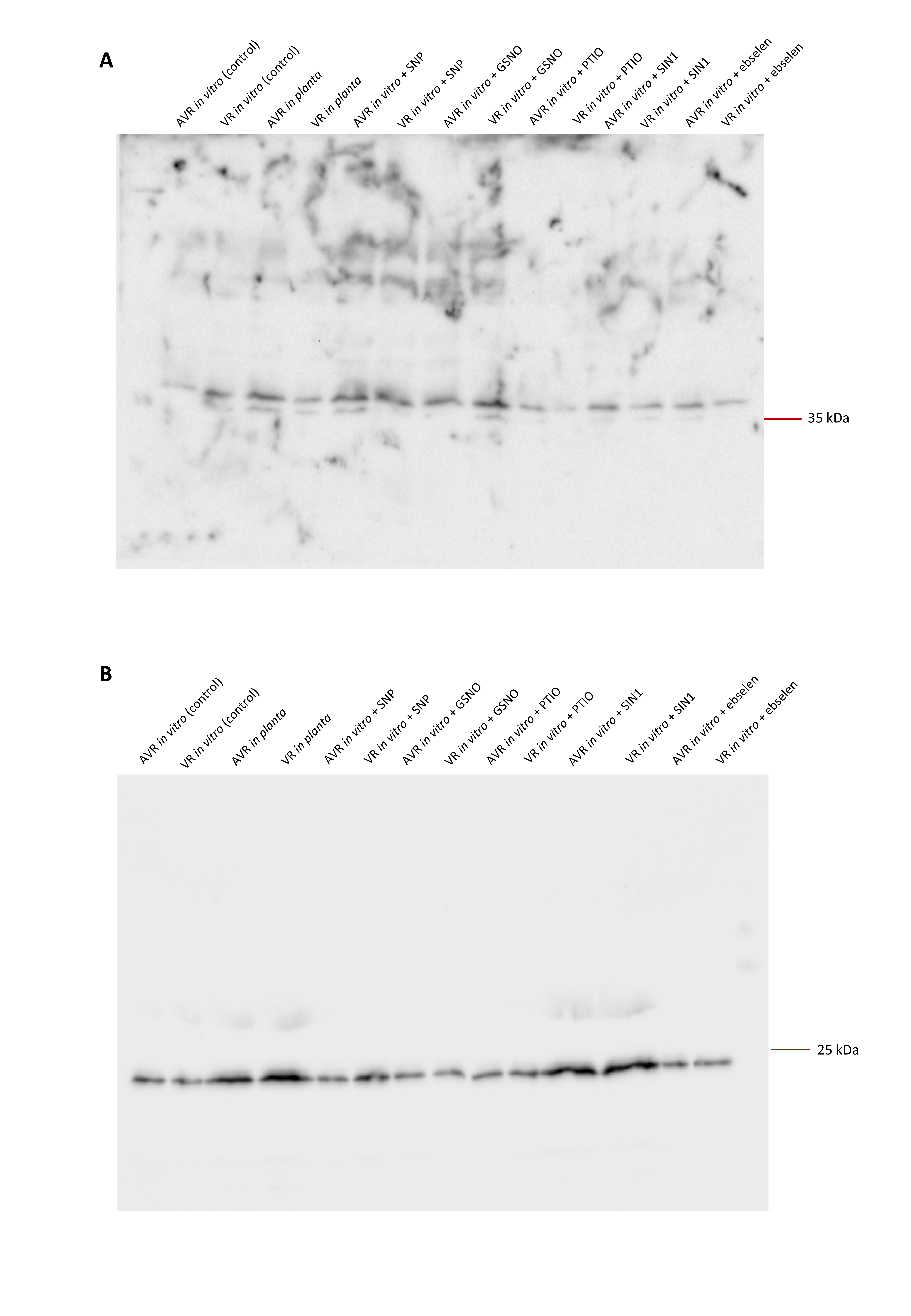

Supplement: Supplementary Figure 4 — Representative Western blot of (A) PiNOD and (B) PRX2 protein accumulation. [file Image_4.tif]

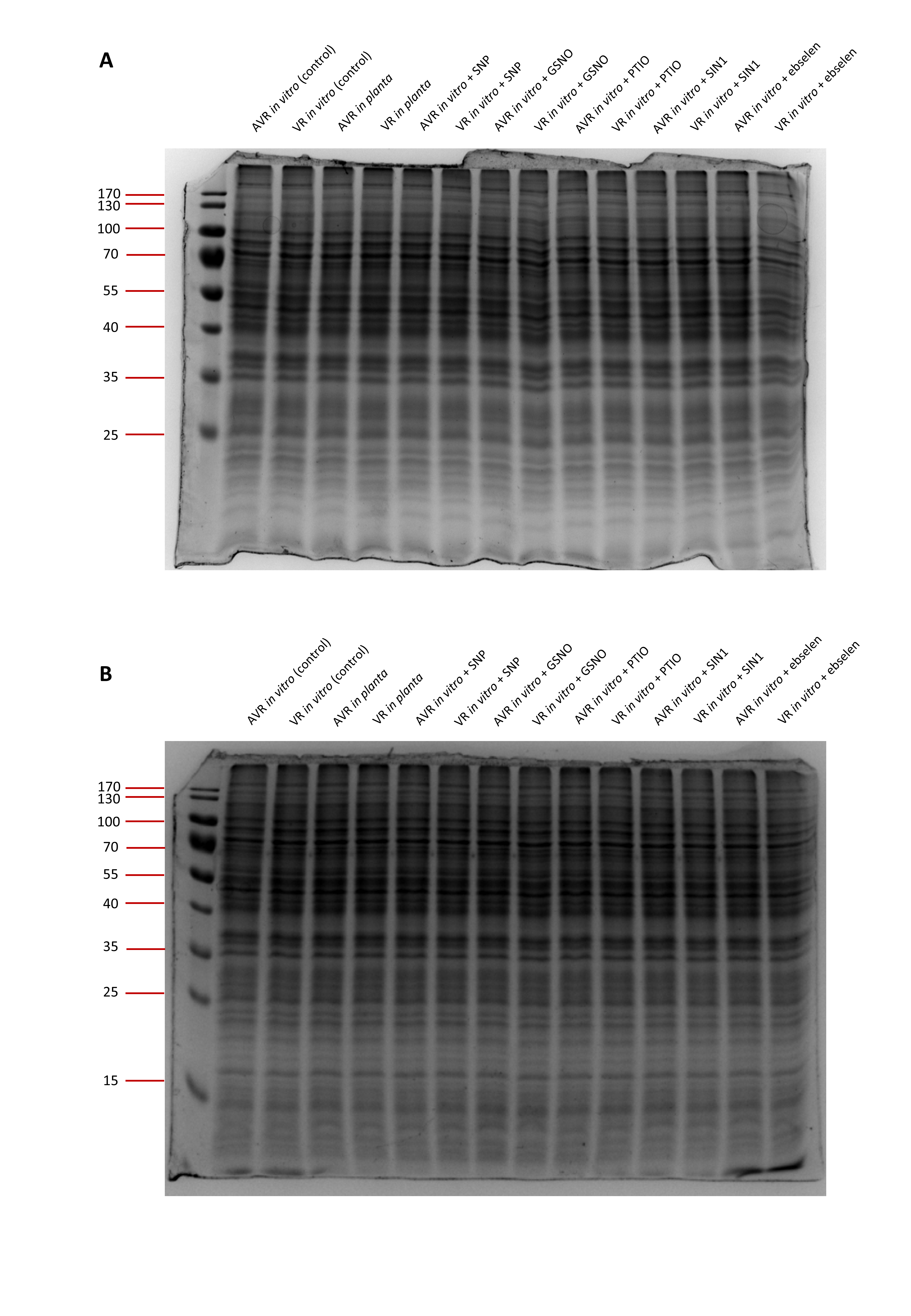

Supplement: Supplementary Figure 5 — Representative SDS-PAGE of proteins stained with Coomassie Brilliant Blue preformed in parallel during (A) PiNOD and (B) PRX2 immunodetection. Fifteen micrograms of protein of each sample was loaded per lane. [file Image_5.tif]
