# Supplementary material for: Insight into metabolic sensors of nitrosative stress protection in Phytophthora infestans
Source: Front Plant Sci. 2023 Jul 20;14:1148222. doi: 10.3389/fpls.2023.1148222 (PMC10399455; doi:10.3389/fpls.2023.1148222)
Supplement: Supplementary Table 1 — Sequences of primers used for the real-time PCR reaction. [file Table_1.docx]

| **Gene number in NCBI base** | **Primer sequence** | **GEN** | **Encoded protein** |
| --- | --- | --- | --- |
| XM_002901230.1 | F: GGACGCCTTTCCTTCCTTCA  R: CTCTGGTGGCCGTCTGTAAG | S3a | 40S ribosomal protein S3a |
| XM_002909124.1 | F: ATGGCTCCCAACCAACAGAC  R: CACCAAGCCAGTTCGAGACT | Pi-NOD1 | Nitric oxide dioxygenase |
| XM_002898388.1 | F: CTTTACCTTCGTGTGCCCCA  R: AGGTGCGAGAACTTGGAGTC | PRX2 | Peroxiredoxin-2 |
| XM_002909471.1 | F: CGCTCCATCCAGATCAACGA  R: CTTCCAGTTGGCAGGACACA | PRX4 | Peroxiredoxin-4 |
| XM_002998982.1 | F: TCCGATCCAGGAGGTGCTTA  R: GCACTCGAACGTGTAGTCCA | ADH3 | Alcohol dehydrogenase class-3 |
